# Moral Concepts Set Decision Strategies to Abstract Values

**DOI:** 10.1371/journal.pone.0018451

**Published:** 2011-04-01

**Authors:** Svenja Caspers, Stefan Heim, Marc G. Lucas, Egon Stephan, Lorenz Fischer, Katrin Amunts, Karl Zilles

**Affiliations:** 1 Institute of Neuroscience and Medicine (INM-1, INM-2), Research Centre Jülich, Jülich, Germany; 2 JARA-BRAIN, Jülich-Aachen Research Alliance, Jülich, Germany; 3 Department of Psychiatry, Psychotherapy, and Psychosomatics, RWTH Aachen University, Aachen, Germany; 4 Section Neurological Cognition Research, Department of Neurology, RWTH Aachen University, Aachen, Germany; 5 INEKO, Department Psychology, University of Cologne, Cologne, Germany; 6 Department of Business Studies – Leadership and Organization, FernUniversität Hagen, Hagen, Germany; 7 Institute of Economic and Social Psychology, University of Cologne, Cologne, Germany; 8 C. and O. Vogt Institute for Brain Research, Heinrich-Heine-University Düsseldorf, Düsseldorf, Germany; Institute of Automation, Chinese Academy of Sciences, China

## Abstract

Persons have different value preferences. Neuroimaging studies where value-based decisions in actual conflict situations were investigated suggest an important role of prefrontal and cingulate brain regions. General preferences, however, reflect a superordinate moral concept independent of actual situations as proposed in psychological and socioeconomic research. Here, the specific brain response would be influenced by abstract value systems and moral concepts. The neurobiological mechanisms underlying such responses are largely unknown. Using functional magnetic resonance imaging (fMRI) with a forced-choice paradigm on word pairs representing abstract values, we show that the brain handles such decisions depending on the person's superordinate moral concept. Persons with a predominant collectivistic (altruistic) value system applied a “balancing and weighing” strategy, recruiting brain regions of rostral inferior and intraparietal, and midcingulate and frontal cortex. Conversely, subjects with mainly individualistic (egocentric) value preferences applied a “fight-and-flight” strategy by recruiting the left amygdala. Finally, if subjects experience a value conflict when rejecting an alternative congruent to their own predominant value preference, comparable brain regions are activated as found in actual moral dilemma situations, i.e., midcingulate and dorsolateral prefrontal cortex. Our results demonstrate that superordinate moral concepts influence the strategy and the neural mechanisms in decision processes, independent of actual situations, showing that decisions are based on general neural principles. These findings provide a novel perspective to future sociological and economic research as well as to the analysis of social relations by focusing on abstract value systems as triggers of specific brain responses.

## Introduction

Research on value systems is of interest in disciplines such as psychology, sociology, socioeconomics, and related fields. Abstract values represent persons' concepts serving as a general framework for any evaluation preceding decisions and actions [1]–[3]. Based on the pioneering work of Piaget [4] and Kohlberg [5] on value research in its present form, two lines of value theories emerged: Value typologies provide different dimensions on which values are based [2]–[3], [6]–[7], without any hierarchical ranking. One of the most robust dimensions is ‘individualism’ vs. ‘collectivism’ [6]–[8]. Individualists are understood as persons, who prefer an egocentric strategy by exerting their own strengths and abilities for personal success, whereas collectivists rely on an altruistic strategy, relationships to other people, and ranking obligations and duties higher than their personal needs. Hierarchical theories rank values according to their importance for the individual or to the complexity of the values [1], [5], [9]. As a synopsis of these two opposing positions, a third line emerged which integrates typological and hierarchical concepts. It states that different hierarchies of values exist in parallel, between which subjects shift depending on their social and professional situation [10]–[11].

Independent of a particular value theory, it is widely accepted that values and personal ideals influence a person's mindset and behaviour. Neuroscience touched this topic by investigating the neural correlates of moral judgement and morality [12]–[14], primarily assessing decision processes in actual dilemma situations. These studies assessed how people decide between two options in a morally challenging situation. Here, brain areas within the frontal and cingulate cortex were found to be involved. The abstract value system of the person, however, was not investigated. Instead, the persons' value system was assessed indirectly, using actual situations in which a normal person would weigh the possible alternatives with respect to the competing moral values. But moral judgement in general should involve a broader range of values as stated in different value theories [1]–[9], and should be relevant not only to moral dilemma, but also to most decisions in every day life [1], [15]. Thus, it might be expected that principles of decision making found in actual moral dilemma situations only show one aspect of a moral general decision principle in humans which is based on each person's value concept.

Thus, assessing such an influence of an abstract value system on human behaviour should address the neural processing of concepts independent from an actual situation [12]. Dealing with abstract values might involve comparable brain areas as recruited in moral judgement tasks, such as the dorsolateral prefrontal, medial frontal, and anterior to midcingulate cortex. But it remains elusive how activation in these brain regions might be modulated depending on different moral concepts in different persons.

In a functional magnetic resonance imaging (fMRI) study on word pairs representing abstract values, we assessed the question how a person's mindset and thus, his or her way of decision making is influenced by the person's predominant value profile. We could indeed reveal differential neural strategies in different persons.

## Results and Discussion

### Behavioural analysis

**Figure 1:**
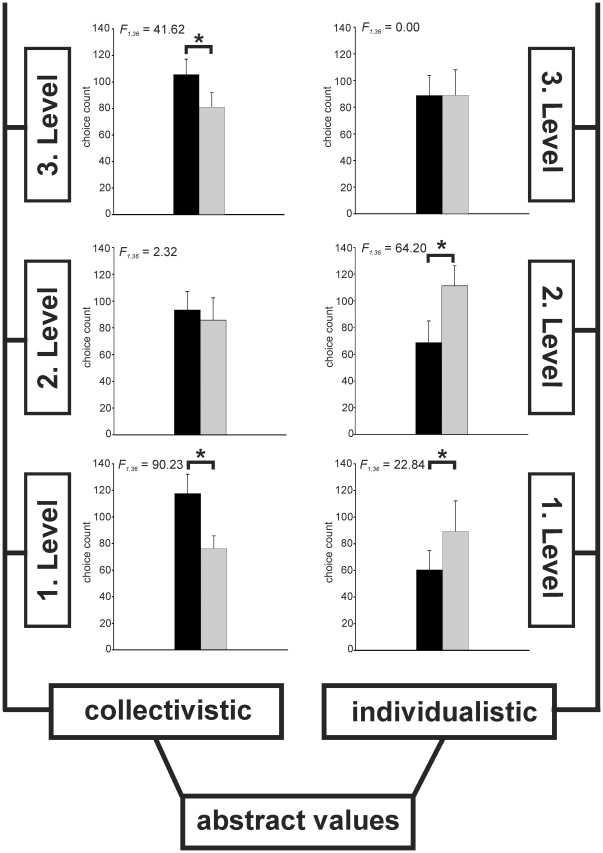
Categories of values as obtained from the value theories. Bar graphs show for each of the six categories the mean count of choices made by the subjects in the MR scanner, averaged over the two groups (Individualists: grey bars, Collectivists: black bars) derived from the two-step cluster-analysis. Error bars provide the standard deviation. Significant differences between groups are indicated by asterisks (ANOVA for interaction between factor ‘group’ and ‘value orientation of stimulus’, *P*<0.001, *df*  = 1, individual *F*-values within figure).

We performed functional magnetic resonance imaging (fMRI) in 38 healthy subjects (21 male, 17 female). Stimuli were visually presented words representing abstract values at different levels of complexity (Fig. 1, Table 1), based on the integrating value theories [10]–[11].

**Table 1:** Stimulus words used for the fMRI paradigm (six categories, six words each).

collectivistic	1. level	2. level	3. level
	(context of family)	(context of peer group)	(context of mankind)
	‘Zusammengehörigkeit’	‘Sicherheit’	‘Menschlichkeit’
	*togetherness*	*safety*	*Humanity*
	‘Geborgenheit’	‘Sorgfalt’	‘Harmonie’
	*protection*	*diligence*	*harmony*
	‘Familie’	‘Loyalität’	‘Gemeinschaft’
	*family*	*loyalty*	*community*
	‘Tradition’	‘Verantwortung’	‘Teamfähigkeit’
	*tradition*	*responsibility*	*teamwork*
	‘Zusammenhalt’	‘Gerechtigkeit’	‘Konvention’
	*solidarity*	*fairness*	*convention*
	‘Beständigkeit’	‘Maßstäbe’	‘Geselligkeit’
	*constancy*	*standards*	*sociability*

individualistic	1. level	2. level	3. level
	(context of self)	(context of peer group)	(context of mankind)
	‘Spaß’	‘Erfolg’	‘Flexibilität’
	*fun*	*success*	*flexibility*
	‘Kreativität’	‘Selbständigkeit’	‘Wertschätzung’
	*creativity*	*autonomy*	*esteem*
	‘Macht’	‘Kompetenz’	‘Unabhängigkeit’
	*power*	*competence*	*independence*
	‘Status’	‘Leistung’	‘Nachsicht’
	*status*	*performance*	*indulgence*
	‘Respekt’	‘Risikobereitschaft’	‘Hingabe’
	*respect*	*risk-taking*	*commitment*
	‘Herausforderung’	‘Zielstrebigkeit’	‘Selbstentfaltung’
	*challenge*	*determination*	*self-development*

The stimulus words in the table are given as the original German word (in single quotation marks) and as the English translation beneath (in italics). Words and their ordering are based on the open systems theory of values [10]–[11] and related theories [1]–[9].

Each word was assigned to one of two types of values, ‘individualistic’ (e.g., ‘power’, ‘autonomy’) and ‘collectivistic’ (e.g., ‘tradition’, ‘community’), each of which encompassed three levels of increasing complexity. The hierarchy of complexity started with a first level of values relevant to family and self, followed by a second level with reference to the peer-group of a person, and reached the third level with values related to mankind (Fig. 1, Table 1). Stimuli were presented as pairs of words from different or the same levels and types, giving a total of 540 trials. Subjects were instructed to spontaneously select the most appealing word in each word pair by button press (forced-choice situation).

Subjects responded in nearly 100% of the trials (mean of missed trials: 6 out of 540). The profile of choices was analysed for each subject to test whether persons could generally be differentiated into groups with differing value preferences. Using a two-step cluster-analysis, subjects were assigned to two groups, one with preference of ‘individualistic’ values (IND; *n* = 14 subjects; 10 male), and the other with preference of ‘collectivistic’ values (COL; *n* = 24 subjects; 11 male). In a 2 (value orientation of group) x 2 (value orientation of stimulus) ANOVA, groups differed significantly (all *P*<0.001) in their choices for first and third level words of the collectivistic type, and first and second level words of the individualistic type (Fig. 1). Groups did not differ with regard to their age and IQ (Table 2), neither overall or with sex as covariate. Since a correlation between personal ideals and personality structure was discussed controversially in different value theories [1]–[11], all subjects were tested on a five-dimensional personality scale (NEO-FFI). Individualists and collectivists only differed significantly in the dimension ‘Conscientiousness’, with collectivists scoring higher on this dimension (Table 2).

**Table 2:** Characteristics of groups COL and IND with regard to age, sex, and personality structure.

	Group IND	Group COL	*P-Values*	*F-Values*
**Age and IQ (standardized data, μ** = **100, σ** = **15)**				
N males	10	11		
N females	4	13		
age ± SD	35.60±12.93	37.91±13.82	*0.34*	*F_1,35_* = *0.96*
age male ± SD	36.55±12.72	43.10±13.60	*0.25*	*F_2,35_* = *1.44*
age female ± SD	33.00±15.12	33.92±13.11		
IQ ± SD	124.33±9.88	120.35±10.55	*0.23*	*F_1,35_* = *1.51*
IQ male ± SD	125.40±9.84	118.20±13.25	*0.48*	*F_2,35_* = *0.76*
IQ female ± SD	122.25±11.15	122.00±8.09		
**Personality structure (standardized data, range 0–4)**				
Dimension ‘Neuroticism’	1.60±0.66	1.53±0.65	*0.76*	
Dimension ‘Extraversion’	2.36±0.42	2.34±0.53	*0.89*	
Dimension ‘Openness’	2.60±0.54	2.31±0.50	*0.10*	
Dimension ‘Agreeableness’	2.49±0.41	2.76±0.47	*0.08*	
Dimension ‘Conscientiousness’	2.71±0.49	3.01±0.38	*0.04* *	*F_1,36_* = 4.65, Wilks' λ = 0.87

All data are given as mean ± standard deviation (SD). Scores on the intelligence quotient (IQ) were derived from the culture-free test CFT-20 [83], scores on the five personality dimensions were derived from the NEO-FFI [84]. Testing for statistical significance was performed using a MANCOVA (age, IQ), and discriminant analysis (NEO-FFI). Significant results are indicated by an asterisk.

**Figure 2:**
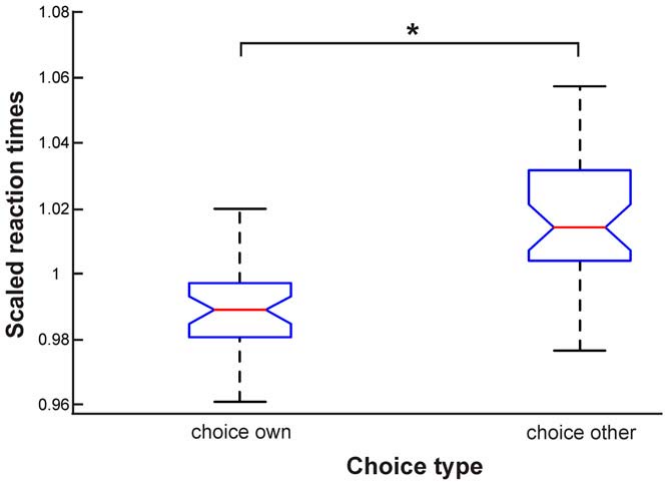
Results of the statistical analysis of scaled reaction times of different trial types. Box plots show mean scaled reaction times with percentiles for the two choice types choice for a word in accordance with one's own value profile (choice own), and choice for a word not in accordance with the own value profile (choice other). ANOVA (*P*<0.0001) revealed a significant effect of factor ‘choice type’ as marked by the asterisk.

The groups as revealed by the two-step cluster analysis represent a distinction in accordance with the value theories, showing a subdivision of subjects on the typological dimension ‘individualism vs. collectivism’. Thus, based on the value theories, one would expect reaction times to differ between the stimuli. In his value study, Graves [11] showed that subjects would react faster to stimulus words in accordance with their own mindset than to words which do not belong to their own mindset. Thus, we analysed the reaction times (RTs) of the subjects by dividing the respective trials into those where subjects chose a word according to their own overall value profile, and those where subjects chose a word not representing their overall value profile. RTs were scaled for each subject individually by the mean RT across all trials since RTs differed considerably between subjects. Scaled RTs then entered an ANOVA to test whether RTs differed significantly for the above mentioned choice types. ANOVA was significant at *P*<0.0001 (*F_1,48_* = 45.46) for factor ‘choice type’. Figure 2 shows the respective boxplots for both choice types, and highlights the fact that RTs for choices not in accordance with the person's overall value profile are significantly longer than those for own words. Thus, subjects indeed acted as predicted by the value theory [11] since decisions against their overall value profile took longer.

It has to be noted that a subdivision of subjects based on the typology dimension ‘individualism vs. collectivism’ was the only statistically testable distinction. Further subdivisions with regard to the different levels of complexity (i.e., the hierarchical element of the value theories) could not be reliably established. Therefore, the following analyses of group fMRI data are based on this result of the two-step cluster analysis, i.e. a subdivision of participants into individualists and collectivists. Such a subdivision of subjects is in line with our presumptions of the integrating value theories. This prerequisite provides the relevant basis for the interpretation of the neurobiological correlates.

### Differences in brain activation between individualists and collectivists

How is this behavioural differentiation of value preferences represented in the brain? Based on the behavioural characteristics of collectivists and individualists as provided by the value theories [1]–[11] it could be hypothesized that collectivists would weigh the given opportunities, also taking their possible repercussion to other people into account, whereas individualists might be more self-centred when making their choice, only bearing in mind the repercussion of their decision on themselves.

The fMRI data of all subjects were analysed for a main effect of factor ‘group’ to identify overall differences in brain activity between individualists and collectivists. Both groups recruited the brain network for reading [16]–[17] (Broca's area [areas 44, 45], posterior inferior temporal gyrus, and occipito-temporal transition on the fusiform gyrus).

**Figure 3:**
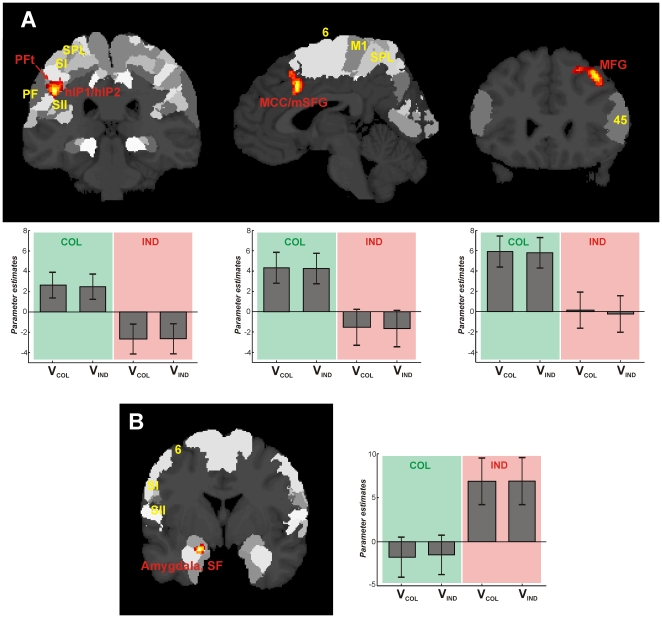
Significant brain activations for the main effects of factor ‘group’. (A) Main effect COL > IND: Coronal and sagittal sections of the MNI single subject template, showing significant activation (*p*<0.05 cluster-level corrected, extent threshold *k* = 200 voxels), labelled in red) within left rostral inferior parietal lobule (PFt) [18]–[19] and intraparietal sulcus (hIP1, hIP2) [20], cluster size: 833 voxels, *T_210_* = 4.45, peak MNI coordinates: *x* = −46, *y* = −32, *z* = 33); right middle cingulate cortex (MCC, BA24) [21] at the border to medial superior frontal gyrus (mSFG), cluster size: 285 voxels, *T_210_* = 4.16, peak MNI coordinates: *x* = 3, *y* = 16, *z* = 33; right middle frontal gyrus (MFG), cluster size: 577 voxels, *T_210_* = 4.16, peak MNI coordinates: *x* = 39, *y* = 26, *z* = 42. (B) Main effect IND > COL: Coronal section of the MNI single subject template, showing significant activation (*p_uncorr_*_._ <0.001, extent threshold *k* = 10, labelled in red) within the superficial part of the left amygdala (SF [22]), cluster size: 61 voxels, *T_210_* = 4.02, peak MNI coordinates: *x* = −20, *y* = −2, *z* = −21. For reading convenience, surrounding areas of the Jülich-Düsseldorf cytoarchitectonic atlas [23] as displayed by the SPM anatomy toolbox [24] are labelled in yellow whereas areas found to be active in the present study are labelled in red. Yellow labelled area codes are as follows: hIP1/hIP2: areas of anterior intraparietal sulcus, M1: primary motor cortex, area PF: area of rostral inferior parietal lobule, SI: primary somatosensory cortex, SII: secondary somatosensory cortex, SPL: superior parietal lobule, area 6: premotor cortex. Bar plots beneath (for A) and beside (for B) each section show the parameter estimates (i.e. the strength of the BOLD-effect for each condition as measured during fMRI, revealing if and to what degree the each condition contributed to the observed activation) at peak MNI coordinates for collectivists (COL; green), and individualists (IND; red) when choosing either individualistic (V_IND_) or collectivistic values (V_COL_). Error bars provide the standard error.

But the general processing strategies on all decisions (either congruent, i.e. collectivists chose collectivistic values and individualists chose individualistic values, or incongruent, i.e. collectivists chose individualistic values and vice versa) differed between groups (Fig. 3): Collectivists showed significantly stronger activation (main effect COL > IND) within left rostral inferior parietal cortex (IPL, area PFt [18]–[19]) and intraparietal sulcus (IPS, areas hIP1, hIP2 [20]), the right midcingulate cortex (area 24; MCC [21]) at the border to the medial superior frontal gyrus (mSFG), and the right middle frontal gyrus (MFG). Conversely, individualists showed a significantly stronger activation (main effect IND > COL) in the superficial part of the left amygdala (area SF [22]).

Whereas collectivists recruited a network of cortical brain areas, individualists showed stronger activation of a subcortical structure. Such differential recruitment of cortical vs. subcortical structures points to fundamentally different strategies of individualists and collectivists when facing decisions. This is even more important when considering that these structures belong to different systems, i.e. the amygdala to the limbic system (for the individualists) and frontal and parietal areas to association cortices (for the collectivists). The following paragraphs should elucidate on the basis of the existing literature how these neurobiological correlates might reflect differential ways of thinking for persons with different moral concepts as hypothesized based on the value theories.

Collectivists recruited three different cortical brain regions during their decisions. Characterizing the different contributing areas of the network would provide a cue on how these areas might be used in collectivists to reach a decision. If there exists a neurobiological correlate for the value-theory driven hypothesis that collectivists would weigh the given alternatives, especially with regard to an acceptable outcome for others, one would expect at least two different requirements to be fulfilled: (i) ability to weigh alternatives with regard to their outcome (such as detection of potential failures or bad options), and (ii) appreciation of others with judgement about their needs. The possibility to fulfil these requirements should therefore be provided by areas of the recruited cortical brain network.

One area recruited by the collectivists was the left IPL/IPS region, which has been implicated in non-spatial stimulus selection. According to Mevorach et al. [25], during stimulus selection, the left IPL/IPS provides a top-down control of extrastriate visual areas to regulate the processing of non-salient stimuli, thus enabling the subject to ignore salient aspects and choose non-salient stimuli [26]. The effect does not seem to reflect task difficulty, since no increased activation in left IPL/IPS was found when the task was simply made more difficult without a corresponding change in saliency [27]–[28]. Based on these former studies, the recruitment of the left IPL/IPS by the collectivistic group could be interpreted as enabling the person, for each word pair, to reject the possibly at first most salient word. Instead, collectivists were also able to appreciate the less salient word and choose it. It has to be noted that, in the present study, such a saliency effect could be observed on abstract value words, not objects as in former studies [25]–[28]. This might provide further hints that this effect is a more general principle which only was assumed so far [26].

The MCC was linked to error detection and response selection [29]–[30], aiming at avoidance of a bad outcome [31]. Thus, behaviour will be reorganized to promote actions which can effectively avoid future harm. This theory of MCC function was originally based on pain and distress studies [32]–[33], but later also established for other kinds of cognitive processing with the need for avoiding a bad outcome [34]–[35]. In meta-analyses, it was furthermore stressed that especially this part of the cingulate cortex forms the cognitive division, being activated in cognitively demanding tasks. This could involve motor-response selection tasks, tasks with divided attention or with competing streams of information [36]–[37]. Especially for the intersection between MCC and mSFG, as found in the present study, the concept of counterfactual thinking has lately been proposed [38]. This concept enables the person to ask what would have happened if the decision had been the other way round. The involvement of the mSFG particularly refers to counterfactual reasoning about action versus inaction. Here, the mSFG serves as an internal action monitor, which also includes the suppression of a prepotent action or monitoring the outcome of a self-selected action [38]–[41]. Thus, the activation of the present study could likely be interpreted as serving as a “response monitor” for the selection process required when choosing between two abstract values. But the mSFG activation could furthermore play a role in the social context of the decision process. It was reported that mSFG was involved in forming judgements about other people, especially concerning the reputation a person has in view of another [42]–[44]. Being only activated in the collectivists, they seem to use this cortical region to carefully weigh their possibilities to reach the best possible solution with the best outcome for them and for others, also taking care of their reputation.

The MFG was found to be active during self-other differentiation processes, enabling the subject to ascribe a mental state to another person in relation to one's own [45]. As part of the dorsolateral prefrontal cortex (BA 46 and 9), this region seems to be involved in social reasoning. It was shown that the MFG plays a role in the evaluation of the fairness and permissibility of behaviour as demonstrated by fMRI and transcranial magnetic stimulation neuroeconomical studies [46]–[48]. This involvement in socially relevant decisions was further supported by studies in which social norms were violated, pointing to a respective evaluative function of the right MFG in particular [48]–[50]. In moral dilemma situations, this region is also involved, assumed to provide the normative evaluation when different moral goals conflict with each other [51]. Thus, the involvement of the MFG in the present study could be interpreted as being the “social monitor”, comparable to the “response monitor” of the MCC/mSFG region, in a situation where collectivists had to decide between different abstract moral values. Especially the fairness and social permissibility aspect might be essential for the collectivists, deduced from their orientation towards other people. Even when deciding in an abstract fashion, collectivists seemed to try to find the fairest solution when making their choice between two abstract values. Therefore, again the present study points to a more general principle of socially relevant decision-making, irrespective of an actual situation.

Interpreting the possible role of this cortical brain network recruited by the collectivists during the decision process, their strategy might likely be called a ‘balancing and weighing strategy’. This was hypothesized based on the behavioural characteristics of the collectivists. The recruited brain areas contribute different aspects of this strategy, since they enable the collectivists to weigh both alternatives and try to detect possible errors or any social unfairness in their decision, aiming at finding the optimal choice for everyone. Together, these areas form a cortical brain network which is recruited by the collectivist to apply their orientation towards other people with an altruistic attitude to decision processes, underpinning our theory-driven hypothesis of how a neurobiological correlate of a collectivistic moral concept might be organized to reach a decision.

For individualists, on the contrary, a different strategy would be hypothesized based on their behavioural characteristics. According to the value theories, individualists would most likely focus the outcome of their decision to their personal advantage or benefit. The potential neurobiological correlate of such a strategy was at least completely different from the one of the collectivistic strategy, i.e. the involvement of a subcortical limbic structure in contrast to a network of cortical association regions.

The only activation found to be more active in the individualists than the collectivists was the superficial part SF of the left amygdala. The amygdala was implicated in processing of stimuli which are either arousing or emotional. Here, the emotional valence could have been either positive or negative [52]–[56]. Spoken in a more general fashion, the amygdala seems to process the relevance of a stimulus in a personal situation. Ascribing this role to the amygdala might point to possible differential response mechanisms of the amygdala in different people, depending on their interpretation of the situation [55]–[56]. The preponderance of amygdala activation in only one of two groups of people in the present study, i.e. the individualists, supports this theory. It shows that a person's mindset and general value orientation might be one factor which influences their point of view and consecutively, the response characteristic of the amygdala. The specific activation of only the superficial part SF of the amygdala further supports the current interpretation of the amygdala providing the social information within the decision process of the present study. This SF region was found to be important for continuous evaluation of socially relevant situations [57]–[58]. Having found activation only in the left amygdala seems to further support the so far proposed interpretation: In two meta-analyes the left amygdala was found to be not only involved in pure negative emotion processing, but furthermore in a sustained evaluation process of the emotional valence and arousal of the stimulus [59]–[60].

Thus, the strategy of the individualists might be interpreted as a ‘fight-and-flight’-strategy. They did not try to weigh each decision in each possible way as the collectivists did, but aimed at detecting the social relevance, and consecutively, the possible menace of the decision with regard to their own social status. Behaviourally, this is in accordance with the individualists' orientation to egocentric values. Based on the value theories, it was assumed that individualists would focus on their personal outcome when facing a decision. The neurobiological correlate of such a strategy found in the present study supports this notion, but also reveals the fundamental difference to the strategy of the collectivists: individualists seemed to be more emotionally engaged in a decision process, entering a different level of processing than collectivists.

It has to be stressed that these differential strategies were found in decisions on *abstract* moral values, irrespective of an *actual* situation, in contrast to the referenced literature. Thus, moral concepts and general value orientations provide principle brain mechanisms for the subject of how to approach a decision. Here, brain regions beyond the known cingulate and prefrontal regions which are recruited during actual moral conflict situations [12]–[15] were involved.

Taken together, these findings could support the notion of two main components existing within the complex of ‘morality’: moral reasoning (cognition) and moral feelings (emotion), which are supported by different networks of cortical (cognition) and subcortical (emotion) areas [61]–[63]. Together with studies on antisocial and psychopathic behaviour it was argued that either one or the other system might be impaired in antisocial individuals, mainly preventing them from having a feeling for morality (emotion component) [61]. Comparable to that dichotomy, we could hypothesize that the two components of morality, i.e. emotion vs. cognition, are generally demanded differently, depending on the predominant moral concept of a person. Whereas collectivists seem to concentrate on moral reasoning aspects when solving a decision, individualists are more involved with the moral emotion aspect. It can be assumed that in principle, all people have access to both components of moral decision making. But depending on their current moral concept, the one or the other component outweighs the other. The idea of the integrating value theories [10]–[11] that every person is in principle equipped with either moral concept, switching between the different manifestations depending on their social and professional situation, provides the theoretical background for such an interpretation. Thus, our results provide a new aspect to the discussion about the possible dichotomy of moral judgement, showing that even in healthy, psychosocially normal persons, one or the other component (cognition or emotion) might be dominant.

In context of psychopathic behaviour and possible impairments in the neural circuits of morality, it was argued that antisocial behaviour is to occur first, and then causes a switch in moral thinking, not vice versa. This was explained as a need to adjust moral thinking to repeated (antisocial) activities to reduce cognitive dissonance [64]–[66]. With respect to healthy, psychosocially normal persons, a comparable causal system could be assumed: If the moral concept of a person shifts depending on his or her social or professional situation [10]–[11], the predominance of one or the other component of moral judgement might shift sequentially to adjust the decision processes to every day life. On the contrary, it seems unlikely that a shift in the decision making system would precede a shift of the overall moral concept. But for this problem, our results provide only first hints for one of the two possibilities, leaving a further investigation for future studies.

To our knowledge, this is the first study which experimentally investigates neurobiological correlates of how a person's mindset might influence the way of decision making. With our design, we were able to find behavioural data which distinguished subjects based on their overall value profile which provided the basis for consecutive analysis of possible neurobiological correlates. The interpretation of these findings must remain tentative. But based on the insights gained from the present study, showing that subjects can be grouped with regard to their overall value concept, and that neurobiological correlates could be identified for such a distinction, modelling of the second part of psychological value research, i.e. the different levels of increasing complexity could be a challenge for future studies.

### Conflict processing in individualists and collectivists

This difference in processing strategies between individualists and collectivists when facing abstract value decisions lead to the question if these decisions on abstract values also bear a conflict potential which might involve comparable brain areas as found in actual moral dilemma situations. Such conflicts might then be experienced and processed differently in persons with different moral concepts. We tested this hypothesis by taking the non-chosen words in each trial as a possible conflict reason. Thus, the fMRI data of both groups were re-analysed, sorting the trials into non-chosen individualistic and collectivistic words, assuming that the volunteers might have experienced a conflict when they did not choose in accordance with their overall value profile.

A first hint to such conflict situations was provided by the subjects' reports when debriefing them after scanning. Subjects reported that in most trials they easily chose one of the two presented words. But there were also trials in which both words were equally wrong for them, causing the subjects to feel that their choice would be equally bad. Furthermore, there were trials in which both words were equally good, which caused subjects to have a problem with choosing one of them. Finally, there were trials in which subjects experienced that they did not chose in line with the rest of their decisions, a fact which made them feel angry.

To test if this assumption also has a behavioural basis derived from the data during the experiment, we analysed the reaction times (RT) to different stimuli, taking the RTs as an indicator for potential conflict [1]–[11]. Thus, RTs were grouped according to the sorting of the trials, providing four different groups of RTs: 1. both words belonged to the overall value profile of the subject (positive conflict); 2. neither of the words belonged to the overall value profile of the subject (negative conflict); 3. only one word belonged to the value profile of the subject, and the participant chose in accordance with the own value profile (no conflict – positive decision); 4. only one word belonged to the value profile of the subject, but the participant did not choose in accordance with the own value profile (no conflict – negative decision). Based on the value theories it could be hypothesized that trials of group 1 and 4 would cause a potential conflict, because they resulted in not having chosen a word of one's own value profile.

**Figure 4:**
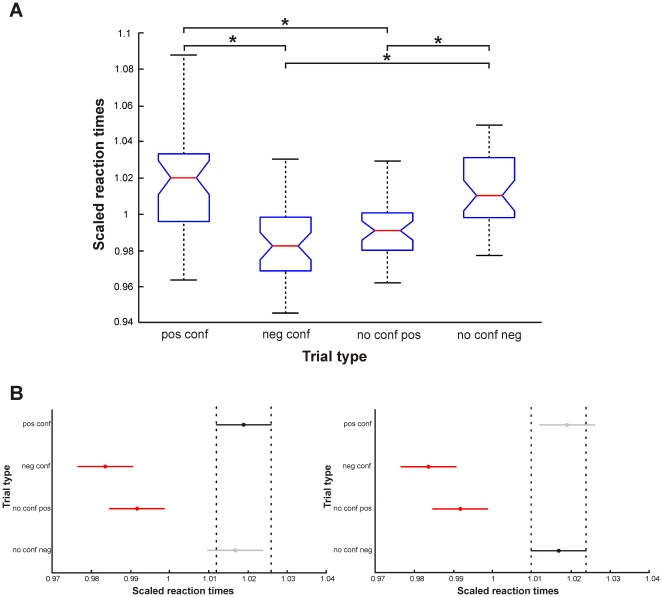
Results of the statistical analysis of scaled reaction times of different trial types. (A) Box plots showing mean scaled reaction times with percentiles for the four trial types positive conflict (pos conf), negative conflict (neg conf), no conflict – positive decision (no conf pos), and no conflict – negative decision (no conf neg). ANOVA (*P*<0.0001) revealed a significant effect of factor ‘trial type’. Asterisks mark those pairwise comparisons which proved to be significant during consecutive multiple comparison testing. (B) Dot plots showing for trial type ‘pos conf’ (left panel, black bar) and ‘no conf neg’ (right panel, black bar) that their reaction times were significantly different from trials ‘neg conf’ and ‘no conf pos’ (red bars), but not from each other (grey bar). Bars mark the standard error of each estimated mean scaled reaction time (marked as dots).

To statistically test this hypothesis, RTs for each trial type were first scaled for each subject individually by the mean RT of each subject because averaged RTs differed considerably between subjects. Scaled RTs entered an ANOVA to test if RTs of the four trial types differed significantly from each other across subjects. The ANOVA was significant at *P*<0.0001 (*F_3,148_* = 21.23) for a main effect of the factor ‘trial type’. Thus, consecutive multiple comparison testing was applied to identify those pairs of trial types for which RTs differed from each other. These tests revealed that RTs of trial types 1 and 4 were significantly longer than those of trial types 2 and 3. This shows that the decision process for trials 1 and 4 took longer than for trials 2 and 3. Together with the reports of the subjects after scanning, this result is a further hint that decisions were experienced differently depending on the trial type, with greater potential for conflict when a word of one own's value profile was not chosen. Statistical results are summarized in Figure 4.

Based on these behavioural peculiarities, we re-analysed the respective brain data to investigate if such behavioural differences have a correlate in brain activity, referring to the different trial types as different potential conflicts which subjects experienced.

**Figure 5:**
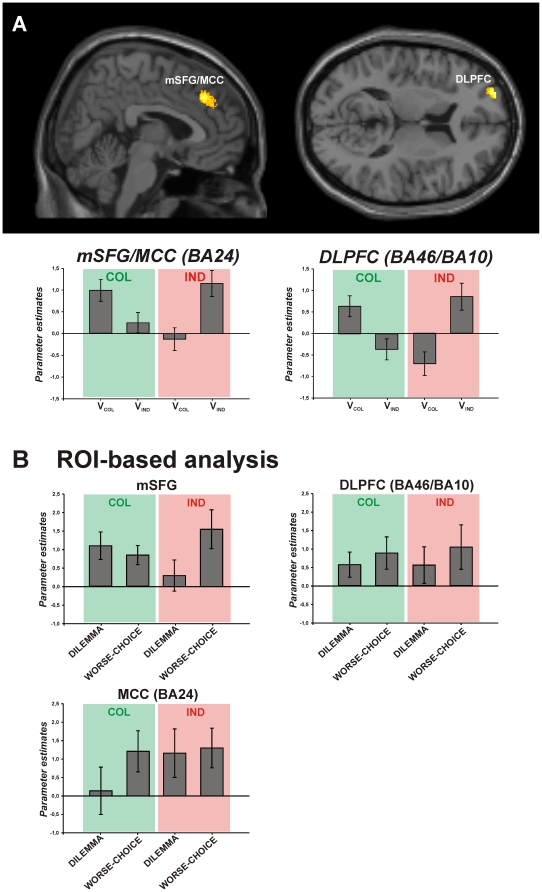
Significant brain activation and consecutive ROI-based analysis for non-selected words. (A) Sagittal and horizontal section of the MNI single subject template, showing significant activation (*p_uncorr_*<0.001, extent threshold *k* = 150) within the dorsolateral prefrontal cortex at the border to the frontal pole region (DLPFC (BA46/BA10), cluster size: 168 voxels, MNI coordinates of peak activity: *x* = −27, *y* = 56, *z* = 15), and the medial superior frontal gyrus (mSFG, cluster size: 315 voxels, maximum 1, MNI coordinates of peak activity: *x* = 6, *y* = 35, *z* = 36) at the border to the middle cingulate cortex (MCC (BA24 [19]), maximum 2, MNI coordinates of peak activity: *x* = 2, *y* = 45, *z* = 30). Bar plots beneath the sections show the parameter estimates (i.e. the strength of the BOLD-effect for each condition as measured during fMRI, revealing if and to what degree the each condition contributed to the observed activation) as in Fig. 2. (B) ROI-based analysis in the same brain regions as in A, beneath the respective section of A. The MCC/mSFG cluster was separated for this analysis by applying a significance threshold of *p_uncorr_*<0.0005 to the statistical map of A, allowing for a separate extraction of parameter estimates at each maximum individually. Each graph shows the parameter estimates of activations for the two incongruent conditions (DILEMMA, WORSE-CHOICE). Error bars provide the standard error.

In this analysis, the brain network for reading was found again. Additionally, a significant interaction effect was found in two brain regions: left dorsolateral prefrontal cortex (DLPFC) at the border region between BA46 and BA10, and right medial superior frontal gyrus (mSFG, maximum 1) at the transition to the midcingulate cortex (area 24, MCC, maximum 2) [21] (Fig. 5).

Activation within these brain regions was driven by those trials in which subjects rejected their predominant value, i.e. collectivists rejected collectivistic and individualists rejected individualistic values (Fig. 5A). Thus, these brain regions were significantly involved when subjects experienced a conflict. Refusing a congruent value was possible either when two ‘wrong’ values constituted a trial (DILEMMA condition), or when one word of each value type was presented but subjects made a ‘wrong’ decision, i.e. not congruent with their dominant value profile (WORSE-CHOICE condition). Thus, a consecutive region of interest (ROI) analysis was carried out to identify the condition which perplexed subjects the most (Fig. 5B): While individualists recruited mSFG only during the WORSE-CHOICE condition, collectivists used this brain region equally in both conditions. A mirror-inverted activation pattern was found in MCC. Within DLPFC, activation did not differ between groups, but was generally higher for the WORSE-CHOICE than for the DILEMMA condition.

Having not chosen in accordance with the own value concept thus indeed caused a conflict and involved comparable brain areas as found in moral dilemma situations [12]–[15]. Here, the DLPFC was ascribed the role of a rule keeper, providing general rules for persons' behaviour in decision processes [67]. This region may interact with other frontal regions [68], such as the mSFG/MCC. Both collectivists and individualists recruited the DLPFC equally strong, with a slight preponderance during the WORSE-CHOICE condition, which might reflect an equally high need for general rules in a decision process. But in individualists, the processing of this conflict was mainly supported by recruitment of the mSFG, showing that the conflict was caused by the counterfactual thinking which reveals that it would have been possible to choose a congruent value in accordance with the own value profile. Taking the social relevance of the mSFG into account as well [42]–[45], this preponderant activation of the mSFG in individualists might again show that they try to do what is best for themselves but perhaps mainly because they want to be seen in a good light by others (i.e. reputation). The WORSE-CHOICE condition might provide a situation in which the individualists fear a loss of their reputation because they chose contrary to their ‘normal’ choices. Collectivists, on the other hand, additionally activated the MCC, trying to detect if there was an error in the decision. This again matches their behavioural characteristics of orientation towards other people.

Thus, the conflict analysis supports the notion of different strategies for individualists and collectivists when facing value-based decisions.

### Conclusion and outlook

The present study demonstrates that persons with different value preferences apply different neural strategies when facing a decision. These neurobiological correlates reflect hypotheses derived from behavioural characteristics of persons with different moral concepts. As shown for decisions independent of an actual situation, the current analysis provides a general basis for the understanding of decision processes in the brain. Brain areas beyond those activated in actual moral dilemma situations were found to be involved. It remains for future studies to elucidate if neural correlates can also be established for other typological or hierarchical characteristics of values, beyond those found here for the value typology ‘individualism vs. collectivism’. Since value theories have also been applied to economics, leadership, and organizational research [69]–[70], including cultural differences [6], understanding of the neurobiological basics of value processing in persons with different value preferences is likely to have a profound impact on future research in these areas.

## Materials and Methods

### Ethics Statement

The experimental setup of the study was approved by the local Ethics Committee of the RWTH Aachen University, Germany. Written informed consent was obtained from all participants.

### Participants

38 healthy volunteers participated in the experiment (21 males, mean age ± SD  = 39.67±13.25, range 22 – 61; 17 female, mean age ± SD  = 33.71±13.11, range 19 – 59). All participants were native German speakers and had normal or corrected-to-normal vision. Subjects had no known history of neurological or psychiatric disorders. One male subject was excluded from the brain data analysis due to failure of pre-processing of the data, thus only being considered for the behavioural analysis.

### Experimental design, stimuli, and stimulus presentation

Each participant performed a functional magnetic resonance imaging (fMRI) forced-choice paradigm on words with value-based meanings. These words were generated based on psychological theories and general concepts of human basic values [1]–[11]. Following these classification of values, two main sections of values could be differentiated: individualistic (i.e. self-centred) and collectivistic (i.e. group-oriented) values. Within these two sections a further differentiation of values is possible with respect to their relation to other individuals, providing an ordering of values referring to increasing complexity: at a first level, the most basic values appear, encompassing only the individual itself and significant others; at the second level, values in relation to peer groups, like colleagues, friends etc., are based; at the third level, values with relation to every other person are grouped. This hierarchical ordering system of values and value development in humans is based on early psychological theories of e.g. Piaget [4], Maslow [9] or Kohlberg [5]. In total, six value categories were used within the current experiment.

For each of the six word categories, six different words were generated based on words provided in value theories [1]–[11]. Since German language is case sensitive concerning nouns (capital initial letters) and verbs or adjectives (small initial letters), it was assured that only nouns were chosen as stimulus words for the paradigm. Verb- or adjective-derived nouns were excluded in order to control for syntactic word category. In order to generate accurate German words with different value meanings, translations of words from these earlier studies [1]–[11] were checked for the most selective synonym using the German Duden glossary of synonyms [71]. This procedure was necessary since direct translation of words from the original publications was not always suitable due to ambivalent meaning in German language. Translations were double-checked for accuracy and appropriateness by speech and language therapists of the Neurolinguistics Department of the RWTH Aachen University. The stimulus words for all categories can be found in Table 1.

Before entering the scanner, participants were instructed on the general design of the task, i.e. participants just knew they would see a set of word pairs, being presented in a rapid sequence. They were instructed to spontaneously choose the word of each word pair which appealed most to them, independent of any actual situation. The participants did not see the words before the start of the experiment in the MR scanner. Explanation about the intention of the study or the content of the stimulus words was not provided to assure impartiality of the participants when performing the task in the MR scanner. To assure that subjects understood the general principle of how to choose words, they were provided with examples from fields other than value concepts, e.g.: “You see the words ‘vanilla flavour’ and ‘chocolate flavour’: Which word appeals most to you, independent of any given situation?” or “You see the words ‘red’ and ‘green’: Which word appeals most to you, independent of any given situation?” Selection of words was indicated by button presses, using the left index finger for the left word on the screen and the right index finger for the right word on the screen.

After scanning, subjects were debriefed of the experiment to ensure that the task was carried out as intended. Therefore, subjects were asked (in accordance to former studies of value research [1]–[11]) to provide a general appraisal of how they experienced the different choice situations.

Word pairs were presented as written strings in Helvetica font at 48 pts, with one word on the left and one word on the right side of the screen, equally distant from the centre of the screen. Each word from each category was combined with each word from every other category, providing a total of 540 word pairs as stimuli. Each word appeared 30 times, 50% of the trials on the left and 50% on the right side of the screen. This was assured not only for the overall appearance of the word across different categories, but also for the combination of the word with six words from one other category. This change in position was implemented in order to avoid habituation effects or possible preferences of the subjects for one side of the screen.

Stimuli were back-projected onto a screen placed on the back wall of the scanner room, seen by the subjects via a small angled mirror suspended from the top of the head coil. Stimulus presentation was controlled by a computer placed in the control room using Presentation software (Neurobehavioral Systems, Albany, CA, USA).

The study employed a modified event-related design. Stimuli were presented in randomized order, with a different randomisation for each participant. The total duration of the experiment was about 22 minutes. Each trial, i.e. presentation of each word pair, lasted 1.3 seconds, followed by a blank screen for 1 second, providing an inter-stimulus interval of 2.3 seconds. The combination of the total trial duration (2.3 s) and the fMRI repetition time (2.5 s; cf next paragraph) resulted in distributed sampling serving as a temporal jitter [72]–[73]. The distributed sampling procedure was chosen instead of a jitter by implementation of a variable time period between each trial onset to ensure equally short trial durations for each and every trial. Such rapid presentation of stimuli was chosen to reliably detect the relevant effect of how values are processed in the brain. According to the value theories [1]–[11], a short presentation of stimulus words is essential to gain an unbiased view of a person's mindset. Otherwise, a potential bias might be introduced if subjects are given too much time to rethink their answer. A further advantage was the increased number of stimuli for each value category presented in a reasonable total time frame, which increases statistical power [74]–[75].

### Functional and anatomical magnetic resonance imaging data acquisition

The functional magnetic resonance imaging (fMRI) experiment was carried out on a 3T Siemens Tim-TRIO scanner (Erlangen, Germany). A standard birdcage head coil was used with foam paddings to reduce head motion. Functional data were recorded from the whole brain, using a gradient-echo echoplanar imaging (EPI) sequence for blood-oxygen-level-dependent (BOLD) contrast with the following parameters: echo time (TE)  = 30 ms, flip angle  = 90°, repetition time (TR)  = 2.5 s, 41 axial slices, slice thickness: 3 mm, slice distance 10%, field of view (FoV)  = 200×200 mm^2^ with an in-plane resolution of 3 mm×3 mm.

After the experimental EPI runs, a high-resolution T1-weighted anatomical image was obtained for later normalisation of the EPI data into MNI space using a 3D-MPRAGE sequence (176 axial slices, TR  = 2.25 s; TE  = 3.03 ms, FoV  = 256×256 mm^2^, flip angle  = 9°, final voxel resolution: 1 mm×1 mm×1 mm).

### Image analysis

Data were processed using MATLAB 7 (The Mathworks Inc., Natick, USA) and the SPM 5 software package (Wellcome Department of Imaging Neuroscience, London, UK, http://www.fil.ion.ucl.ac.uk). Pre-processing of each data set included the standard procedures of realignment, normalisation to the MNI single subject template [76] and spatial smoothing with an 8 mm FWHM Gaussian kernel. The anatomical images served as reference for the transformation to the MNI reference brain, co-registering all functional EPI images to the corresponding anatomical data set, using the unified segmentation approach [77].

For the statistical analysis at the single subject level, trials were assigned to the six word categories individually for each subject, using the subject's decision on each word pair as the categorizing variable (individual selection of trials from each subject's Presentation log-files, providing six trial categories). Failure to choose a word within the time frame of the inter-stimulus interval of 2.3 seconds was counted as a missed trial. The respective trials were excluded from further analysis. The whole study and analysis applied a modified event-related design to optimally model the relevant time periods of such cognitive experiment [78]. Having single events (presentation of each pair of stimulus words as trials), the durations of each trial were set as very short blocks according to the reaction time of the subjects. I.e. the end of each trial was set individually for each trial at the time of the button press, giving variable trial durations. Variable durations for each trial were used in order to model the relevant time period of the BOLD signal during stimulus attainment, cognitive processing of the stimulus, and decision most accurately. Variable trial durations did not enter any further analyses beyond first-level single subject analyses, neither as additional parameters nor as regressors or covariates.

Thus, the relevant block functions resembled such block functions which are known from blocked designs. In the present study, each “block” is in fact a mini-block with duration of several hundred milliseconds (i.e. the reaction time in the individual trial), with steep increase of the slope at stimulus onset, remaining on the activity plateau for the short period until button press (reaction time), and final return to baseline. The duration of the plateau phase was variable, depending on the reaction time to each stimulus. The respective block functions for each category were then convolved with a canonical hemodynamic response function (HRF) with its first derivative to allow for a more flexible and thus optimised fit to the experimental data. According to the Linearity Theory for event-related designs with stimulus-onset asynchrony of around 1 second [79]–[80], overlapping HRFs from consecutive trials (due to rapid sequence of events) could be separated from each other assuming additive effects for the emergent total HRF. For each participant, the contrasts of each category vs. the implicit resting baseline as implemented in SPM were calculated. This implicit resting baseline consisted of all blank-screen intervals between the stimuli. When reaction times were longer than the stimulus presentation time, thus overlapping with the blank-screen period, only the rest of the blank-screen period after the button press was considered for the implicit resting baseline.

For the group analysis, the individual contrast images of all six categories were entered into a repeated-measures ANOVA as a second-level random effects analysis. A factorial design was implemented with factors “subject”, “group” (from the behavioural analysis, either “individualistic” or “collectivistic”), and “trial category”. Coordinates are reported in standard MNI stereotaxic coordinates as implemented in SPM 5 [81].

### Statistical analysis of neuropsychological and behavioural data

Neuropsychological and behavioural data were analysed using SPSS 15 for Windows (SPSS Inc., Chicago, IL, USA).

Behavioural data of subjects' performance during the fMRI experiment were tested to identify sub-groups of the participants related to their value preferences, conducting a two-step cluster-analysis which provides the optimum number of clusters in a given data set (see also [82]). Six variables were entered into the analysis, one for each category of value words. Manifestations of the variables were the count of choices for each participant for each value category, i.e. how often a subject chose a word from the respective value category. The analysis was run allowing for a maximum of 15 clusters, log-likelihood distance estimation, Akaike's information criterion as clustering criterion, no noise-handling for outlier treatment, initial distance change threshold of 0, a maximum of eight branches per leaf node, and a maximum of three depth levels. All variables were standardised during the clustering procedure. A Bonferroni-correction for multiple comparisons was applied. Discriminant analyses were carried out with step-wise inclusion of variables (inclusion criterion of p≤0.05, exclusion criterion of p≥0.10), priors set to equal, and calculation of Wilk's lambda. The correct assignment of participants to one group was tested with the cross-validated statistics, giving a re-classification rate of 100% for both groups.

Outside the scanner, additional neuropsychological data were obtained from each participant. Individual IQ testing was administered using the short form (part 1) of the culture-free intelligence test CFT-20 [83]. Personality traits were assessed using the multidimensional personality inventory NEO-FFI of Costa and McCrae [84], which assesses five robust dimensions of personality (Table 2). These data were used to characterize the resulting groups of the two-step cluster analysis. IQ and NEO-FFI data of subjects were entered into a MANCOVA (for IQ together with age) and a discriminant analysis (NEO-FFI), respectively.
